# Prediction of oxygen uptake in dragon boat athletes using machine learning and multimodal physiological signals

**DOI:** 10.3389/fphys.2026.1740055

**Published:** 2026-09-07

**Authors:** Yongji Yang, Jing Qing, Bing Cui, Xinshi Zhao, Jiabei Yu

**Affiliations:** 1 Department of Physical Education, North China University of Water Resources and Electric Power, Zhengzhou, China; 2 Key Laboratory of Exercise and Physical Fitness, Ministry of Education, Beijing Sport University, Beijing, China; 3 The School of Management and Economics, North China University of Water Resources and Electric Power, Zhengzhou, China; 4 Competitive Sports Research Office 4, Beijing Research Institute of Sports Science, Beijing, China

**Keywords:** dragon boat, machine learning, multimodal signals, oxygen uptake, wearable devices

## Abstract

**Objective:**

This study developed and evaluated a machine learning model for predicting oxygen uptake (VO_2_) in dragon boat athletes using wearable signals and compared performance across signal combinations and device groups.

**Methods:**

Twelve male collegiate dragon boat athletes completed nine tests grouped into six task types (T1–T6): 200-, 500-, and 1000-m all-out paddling tests; an incremental test; a five-stage constant-load test; and four supramaximal-intensity tests pooled as T6. Heart rate, respiratory rate, device-derived minute ventilation, and muscle-oxygenation signals were collected. Fifteen signal combinations (G1–G15) and six device groups (A–F) were evaluated using a multilayer perceptron model. Performance was assessed using mean absolute error (MAE) and root mean square error (RMSE), with mean absolute percentage error (MAPE) also reported.

**Results:**

G13–G15 showed comparable performance, with no significant differences. G14 yielded the lowest MAE and MAPE (2.21 mL·kg^−1^·min^−1^ and 9.48%), whereas G13 yielded the lowest RMSE (3.19 mL·kg^−1^·min^−1^). No significant differences were observed among signal combinations within Groups A, C, D, E, or F. Group F showed the lowest MAPE but did not differ significantly from Group B, whereas Group C showed significantly higher MAPE than Groups B, D, E, and F. Performance did not differ significantly across exercise tasks. During the 1000-m test, predicted and measured VO_2_ were strongly correlated for G15 (*r* = 0.88), with a mean prediction error of 0.44 mL·kg^−1^·min^−1^ and descriptive 95% limits of agreement from −6.32 to 7.20 mL·kg^−1^·min^−1^.

**Conclusion:**

Cardiorespiratory signals provided useful information for wearable-based VO_2_ prediction, whereas the contribution of muscle-oxygenation signals depended on the input configuration. G13–G15 should be regarded as comparably performing configurations, and bilateral muscle-oxygenation monitoring was not consistently superior to unilateral monitoring. Multimodal wearable signals show potential for non-invasive VO_2_ monitoring in dragon boat athletes, although larger samples and independent validation are required.

## 1 Introduction

Dragon boat racing is a high-intensity, rhythmically synchronized team water sport that has significant requirements for the athlete’s aerobic metabolism, cardiopulmonary function, and inter-limb coordination (Ho et al., 2013). Oxygen uptake (VO_2_) is a key indicator of aerobic capacity and overall fitness, reflecting the efficiency of oxygen absorption and utilization during exercise (Bassett and Howley, 2000). In dragon boat training and competitions, real-time monitoring of VO_2_ dynamics provides valuable information for assessing training load, optimizing the use of the energy system and improving sport-specific endurance (Beltrame et al., 2017). For example, the 200 m sprint is mainly based on the phosphogenic and anaerobic glycolytic system, while at distances of 500 m and 1000 m, a greater emphasis is placed on aerobic metabolism (Ho et al., 2013). Thus, real-time monitoring of VO_2_ at different paddling intensities not only helps to shed light on metabolic characteristics at different race distances, but also provides quantitative data to determine training intensity zones (McGuigan, 2017).

Despite the importance of VO_2_ for assessing athletic performance, its practical application in water sports comes with numerous technical difficulties. Traditional methods of assessing metabolism are based on gas analysis systems (for example, MetaMax 3B, COSMED K5) that measure gas exchange parameters in the airways using a breathing mask. However, during on-water or simulated paddling, these systems are subject to movement distortions, hold instability, and environmental noise, leading to signal displacement and measurement bias (Beltrami et al., 2021; DeBlois et al., 2021; McClung et al., 2023). In addition, the use of masks and tubing can interfere with natural paddling mechanics and team synchronization, compromising the ecological validity of the measurements (Barbieri et al., 2020; Beltrami et al., 2021; Tutt et al., 2021). In multi-athlete dragon boat settings, additional factors such as hull vibration, water turbulence, and fluctuating stroke rates further amplify these errors. Therefore, achieving a non-invasive and accurate prediction of oxygen uptake in real training conditions remains an important challenge in exercise-physiology monitoring.

In recent years, the integration of artificial intelligence (AI) and sports science has opened up new possibilities for real-time physiological monitoring using wearable sensors combined with machine learning algorithms (Hossen and Abas, 2025; Hsiao et al., 2025; Sheridan et al., 2025; Zignoli et al., 2020). By equipping athletes with various types of wearable sensors, such as heart-rate sensors, accelerometers, inertial measurement units (IMUs), and near-infrared spectroscopy (NIRS) devices, researchers can continuously record heart rate (HR), muscle oxygen saturation (SmO_2_), device-derived minute ventilation (MV), and respiratory rate (RR) during training. Although these signals do not directly reflect gas exchange, machine learning models can establish nonlinear mappings between these signals and VO_2_ (Ashfaq et al., 2022; Hsiao et al., 2025; Zignoli et al., 2020). Previous studies have shown that deep learning models combining inputs from multiple sources can significantly improve VO_2_ assessment accuracy. Compared to traditional linear regression, such models work better in dynamic and high-intensity conditions, because machine learning algorithms can automatically learn complex nonlinear patterns and adapt to inter-individual variability (Ashfaq et al., 2022; Spathis et al., 2022; Zignoli et al., 2020).

Each of the different types of wearable sensors has its own advantages and limitations. Heart rate sensors are widely used for their convenience and high temporal resolution (Achten and Jeukendrup, 2003), but their accuracy of VO_2_ prediction decreases during high-intensity physical activity or heart rate drift phases (Bent et al., 2020; Gillinov et al., 2017; Spathis et al., 2022). Sports watches worn on the wrist often suffer from movement distortion and non-permanent contact with the skin, leading to delays or noise in heart-rate signals (Gillinov et al., 2017; Martín-Escudero et al., 2023). Conversely, armband-based heart rate monitors provide better adhesion to the skin and resistance to movement, making them more suitable for dynamic water sports (Schweizer and Gilgen-Ammann, 2025; Yu et al., 2023). Meanwhile, NIRS-based muscle oxygen sensors measure local oxygen saturation and deoxyhemoglobin (HHb) concentrations to reflect localized metabolic activity, and their effectiveness has been validated in cycling, rowing, and running (Klusiewicz et al., 2021; Paulauskas et al., 2022; Perrey, 2022). In multimodal fusion frameworks, the combination of heart rate, respiration, and muscle oxygenation signals has been identified as particularly promising for VO_2_ prediction (Ashfaq et al., 2022; Hsiao et al., 2025; Zignoli et al., 2020).

Given the cooperative and symmetric paddling nature of dragon boat racing, the signal acquisition system must ensure high temporal synchronization and low interference. Previous studies have reported that simultaneous bilateral muscle oxygen measurements can better capture whole-body metabolic fluctuations (Paulauskas et al., 2022; Sendra-Pérez et al., 2025). However, few studies have systematically compared how different wearable device configurations affect VO_2_ prediction performance, particularly in hybrid aerobic-anaerobic sports such as dragon boat racing. Most existing research focuses on single-signal sources (e.g., heart rate or power output), overlooking the robustness of multisignal fusion models under dynamic exercise conditions (Sheridan et al., 2025).

Therefore, this study aims to systematically investigate the effects of various physiological signal combinations and wearable device configurations on VO_2_ prediction performance in dragon boat athletes using machine learning models. By integrating multisource physiological data, including HR, RR, MV, muscle oxygen saturation, and HHb and comparing 15 signal combinations and 6 device groups, this study seeks to:

- compare VO_2_ prediction performance across different physiological signal combinations;
- evaluate performance differences among wearable configurations (e.g., heart rate armband, sports watch, and unilateral or bilateral muscle oxygen sensors);
- evaluate the feasibility and application potential of machine-learning-based prediction models in dragon boat performance monitoring.

Given the exploratory nature of this work, which focuses on systematic comparisons across multiple physiological signal combinations and wearable device configurations, the investigation was conducted within a hypothesis-generating framework rather than a confirmatory hypothesis-testing approach.

## 2 Materials and methods

### 2.1 Participants

Twelve male collegiate dragon boat athletes from the Henan Provincial Team, who competed in the 12th National Traditional Games of Ethnic Minorities of the People’s Republic of China, participated in this study. All participants were provincial-level athletes with approximately two years of systematic dragon boat training and a habitual weekly training volume of 14–18 h. The participants included six left-side and six right-side paddlers. Left- and right-side classifications referred to the athletes’ paddling positions in the boat, whereas arm-specific sensor locations were described using the functional terms bottom-hand and top-hand. The athletes’ basic characteristics were as follows (**Table 1**): mean age, 20.5 ± 1.3 years; height, 1.79 ± 0.04 m; body mass, 76.0 ± 4.5 kg; BMI, 23.70 ± 1.02 kg·m^-2^; and VO_2_max, 40.58 ± 3.68 mL·kg^-1^·min^-1^. Inclusion criteria were:

**Table 1:** Characteristics of participants.

Characteristic	Left-side paddlers (*n* = 6)	Right-side paddlers (*n* = 6)	Total (*n* = 12)
Age	20.3 ± 0.8	20.7 ± 1.8	20.5 ± 1.3
Height (m)	1.78 ± 0.05	1.81 ± 0.03	1.79 ± 0.04
Body mass (kg)	74.5 ± 5.5	77.4 ± 3.1	76.0 ± 4.5
BMI (kg·m^-2^)	23.62 ± 1.02	23.77 ± 1.10	23.70 ± 1.02
VO_2_max (mL·kg^-1^·min^-1^)	40.33 ± 4.23	40.83 ± 3.43	40.58 ± 3.68

1. non-smoker status;
2. absence of diagnosed cardiovascular, respiratory, or metabolic disorders;
3. no musculoskeletal injuries or back trauma within the past three months.

All participants provided written informed consent after being fully informed of the procedures and potential risks. The study followed the principles of the Declaration of Helsinki and was approved by the Human Research Ethics Committee of the Department of Physical Education, North China University of Water Resources and Electric Power (20250401038).

### 2.2 Experimental protocol

Each participant completed nine exercise tests, which were grouped into six task types for model development and reporting: a 200-m all-out paddling test (T1), a 500-m all-out paddling test (T2), a 1000-m all-out paddling test (T3), an incremental test (T4), a five-stage constant-load test (T5), and four supramaximal-intensity tests that were pooled and analyzed collectively as T6. The incremental test began at 25 W and increased by 15 W·min^-1^ after a 3-min warm-up until exhaustion (Ho et al., 2013; Singh et al., 1995). The five-stage constant-load test consisted of intensities ranging from 45% to 80% of VO_2_max, with each stage maintained for 8 min. The four supramaximal-intensity tests were performed at >110% VO_2_max on separate days, with a 72-h recovery interval between consecutive tests. Exhaustion was objectively determined when at least three of the following criteria were met (Bertuzzi et al., 2010):

1. an increase in VO_2_ of ≤ 2.1 mL·kg^-1^·min^-1^;
2. post-exercise blood lactate concentration ≥ 8.0 mmol·L^-1^;
3. respiratory exchange ratio (RER) ≥ 1.10;
4. peak heart rate reaching 100% of the age-predicted maximum (220 − age).

All exercise tests were conducted in a fixed order, progressing from lower- to higher-intensity protocols to minimize fatigue carryover and reduce injury risk. A standardized recovery interval of 72 h between consecutive tests was strictly maintained for all participants. All tests were conducted indoors using a dragon boat ergometer (Kayak Pro, Miami, FL, USA) under controlled ambient temperature (20–24 °C) and at least two hours after the participants’ last meal. Before each test, a standardized warm-up was performed. All equipment was installed and calibrated by the same experimenter to ensure measurement consistency.

### 2.3 Data acquisition

Respiratory gas data were collected using a portable metabolic analyzer (MetaMax 3B, Cortex Biophysik, Leipzig, Germany), calibrated with standard gas mixtures (O_2_ 15.0%, CO_2_ 5.0%) and a 3-L syringe for volume verification. VO_2_ was recorded breath by breath and exported from the manufacturer’s software as a 1-H_Z_ time series. No additional resampling, artefact removal, interpolation, or temporal smoothing was applied to the VO_2_ data used for model development and performance evaluation.

Participants simultaneously wore multiple wearable sensors to acquire physiological signals. A smart shirt (Hexoskin, Carré Technologies, Montreal, Canada) was used to record thoracic heart rate (HR_t_), respiratory rate (RR), and device-derived minute ventilation (MV; mL·min^-1^). MV represents the estimated volume of air inspired per minute and was calculated by the device software from the preceding seven complete respiratory cycles. The MV values were exported directly from the Hexoskin software and were not calibrated against a reference spirometer in the present study.

For consistent terminology, the bottom-hand arm was defined as the arm gripping the paddle shaft closer to the blade, whereas the top-hand arm was defined as the arm gripping the T-handle. A heart-rate armband (Polar Verity Sense, Polar Electro Oy, Kempele, Finland) was worn on the upper arm of the bottom-hand side to record heart-rate signals (HR_a_). In addition, a sports watch (Polar Pacer Pro, Polar Electro Oy, Kempele, Finland) was worn on the wrist of the top-hand side to record wrist-based heart rate (HR_w_).

Muscle oxygenation was measured bilaterally using near-infrared spectroscopy sensors (Train Red, Artinis Medical Systems, Nijmegen, the Netherlands), which were placed over the muscle bellies of the biceps brachii on the bottom-hand and top-hand arms to record muscle oxygen saturation (SmO_2_) and deoxygenated hemoglobin (HHb). The sensors employed a source–detector separation of 30 mm, corresponding to an approximate sampling depth of 15–20 mm, allowing assessment of superficial skeletal muscle oxygenation. Adipose tissue thickness was not directly measured and may have influenced signal penetration depth, which represents a limitation of the present study.

All wearable-device data used for modeling were exported at 1 H_Z_. Data from all devices were synchronized using timestamps and common trial-start markers and were subsequently aligned with the VO_2_ time series to ensure temporal consistency across modalities. No additional temporal smoothing was applied after data export.

### 2.4 Signal combinations and device configurations

A total of fifteen signal combinations (G1–G15) corresponding to six wearable device groups (A–F) were designed (see **Table 2**). Each combination integrated different input sources, including HR, RR, MV, and bottom-hand, top-hand, or bilateral muscle-oxygenation signals.

**Table 2:** Signal combinations and wearable-device configurations.

Device group	Signal combination	Wearable-device configuration	Model input variables
A	G1	Heart-rate armband worn on the bottom-hand upper arm	HR_a_
B	G2	Smart shirt	HR_t_, RR, MV
A	G3	Sports watch worn on the top-hand wrist	HR_w_
C	G4	Muscle-oxygenation device on the bottom-hand arm	BH-SmO_2_, BH-HHb
	G5	Muscle-oxygenation device on the top-hand arm	TH-SmO_2_, TH-HHb
	G6	Bilateral muscle-oxygenation devices	BH-SmO_2_, BH-HHb, TH-SmO_2_, TH-HHb
D	G7	Heart-rate armband and bottom-hand muscle-oxygenation device	HR_a_, BH-SmO_2_, BH-HHb
	G8	Heart-rate armband and top-hand muscle-oxygenation device	HR_a_, TH-SmO_2_, TH-HHb
	G9	Heart-rate armband and bilateral muscle-oxygenation devices	HR_a_, BH-SmO_2_, BH-HHb, TH-SmO_2_, TH-HHb
E	G10	Sports watch and bottom-hand muscle-oxygenation device	HR_w_, BH-SmO_2_, BH-HHb
	G11	Sports watch and top-hand muscle-oxygenation device	HR_w_, TH-SmO_2_, TH-HHb
	G12	Sports watch and bilateral muscle-oxygenation devices	HR_w_, BH-SmO_2_, BH-HHb, TH-SmO_2_, TH-HHb
F	G13	Smart shirt and bottom-hand muscle-oxygenation device	HR_t_, RR, MV, BH-SmO_2_, BH-HHb
	G14	Smart shirt and top-hand muscle-oxygenation device	HR_t_, RR, MV, TH-SmO_2_, TH-HHb
	G15	Smart shirt and bilateral muscle-oxygenation devices	HR_t_, RR, MV, BH-SmO_2_, BH-HHb, TH-SmO_2_, TH-HHb

HR_a,_ heart rate measured by the armband; HR_t,_ heart rate measured by the smart shirt; HR_w,_ heart rate measured by the sports watch; RR, respiratory rate; MV, device-derived minute ventilation; BH-SmO_2_ and BH-HHb, muscle oxygen saturation and deoxygenated hemoglobin measured on the bottom-hand arm, respectively; TH-SmO_2_ and TH-HHb, muscle oxygen saturation and deoxygenated hemoglobin measured on the top-hand arm, respectively.

### 2.5 Machine learning modeling

A multilayer perceptron (MLP) regression model was employed, comprising two fully connected hidden layers with 128 neurons each and exponential linear unit (ELU) activation functions. To reduce the risk of overfitting, batch normalization and dropout layers (rate = 0.3) were applied. The output layer contained one linear unit for VO_2_ prediction. The Adam optimizer was used with a mean squared error loss function and an initial learning rate of 1 × 10^−3^.

Separate models were trained for each of the 15 signal combinations under each of the six exercise-task conditions (T1–T6). The four supramaximal trials performed on separate days were combined within athletes and analyzed collectively as T6. An additional Overall model was trained for each signal combination using the pooled observations from T1–T6. Therefore, the Overall performance metrics were generated from models trained using the pooled task data rather than by averaging the six task-specific performance metrics.

Before model training, observations with missing measured VO_2_ values were removed. For non-NIRS predictors, missing values were handled within each athlete using forward and backward filling, followed by median imputation where necessary. Observations missing any NIRS-derived variable required by the corresponding signal combination were excluded. Predictor variables were normalized to the [0, 1] range within each cross-validation fold. The min-max scaling parameters were estimated exclusively from the training-athlete data and subsequently applied unchanged to the held-out athlete.

Training was conducted for a maximum of 100 epochs with a batch size of 32. Early stopping was applied with a patience of 10 epochs, and the model weights associated with the lowest validation loss were restored (Prechelt, 2012). The learning rate was reduced by a factor of 0.10 after five consecutive epochs without improvement in validation loss, with a minimum learning rate of 1×10^−5^. A fixed random seed of 42 was used, and each model–condition analysis was run once without repeated stochastic training runs.

Model performance was evaluated using leave-one-athlete-out cross-validation, implemented through Leave-One-Group-Out cross-validation with the athlete identifier as the grouping variable (Varoquaux et al., 2017). In each fold, observations from all remaining athletes were used for model fitting, whereas all available observations from one athlete constituted the held-out fold. Each athlete with available data was held out once, resulting in 12 folds for most model-condition analyses and 11 folds for conditions in which one athlete did not have usable data.

In the original modeling procedure, the held-out athlete was also supplied as the validation dataset for monitoring validation loss, early stopping, learning-rate reduction, and restoration of the best model weights before final predictions were generated for the same athlete. Consequently, model tuning and fold-level evaluation were not fully independent, and the resulting performance estimates were interpreted as athlete-wise cross-validation estimates rather than estimates obtained from a fully independent test set.

For each signal combination and exercise condition, mean absolute error (MAE), root mean square error (RMSE), and mean absolute percentage error (MAPE) were calculated separately from the predictions generated for each held-out athlete. MAE and RMSE were treated as the primary error metrics because they retain the original unit of VO_2_ and directly quantify the magnitude of prediction errors. MAPE was additionally reported as a scale-normalized percentage measure to facilitate comparisons across signal combinations and exercise tasks. The minimum observed VO_2_ value in the analyzed dataset was 3.12 mL·kg^-1^·min^-1^. Because percentage errors may be inflated when observed VO_2_ values are low, MAPE was interpreted together with MAE and RMSE rather than in isolation. For pooled descriptive summaries, predictions from all available held-out-athlete folds were concatenated, and MAE, RMSE, MAPE, and Pearson’s correlation coefficient (*r*) were calculated from the pooled predictions.

### 2.6 Statistical analysis

All statistical analyses were conducted using SPSS version 24.0 (IBM Corp., Armonk, NY, USA). The athlete, rather than the individual 1-H_Z_ observation, was treated as the statistical unit. For each signal combination and exercise condition, one MAE, RMSE, and MAPE value was calculated from the predictions generated for each held-out athlete. These athlete-level fold estimates were subsequently used in the repeated-measures analyses.

MAPE was used as the dependent variable in the comparisons reported in **Tables 3**–**5** because its dimensionless percentage scale facilitated comparisons across signal combinations, device groups, and exercise tasks. Given its sensitivity to low observed VO_2_ values, MAPE-based comparisons were interpreted alongside the descriptive MAE and RMSE values.

**Table 3:** Bonferroni-adjusted pairwise comparisons of athlete-level Overall MAPE within device groups.

Group	Comparison	n	Mean difference	*SD* of paired differences	Bonferroni-adjusted 95% CI	Adjusted *p*	Cohen’s *d_z_*
A	G1 − G3	12	−0.52	1.52	[−1.49, 0.45]	0.260	−0.34
C	G4 − G5	12	−0.28	5.15	[−4.47, 3.91]	1.000	−0.05
	G4 − G6	12	2.09	2.93	[−0.29, 4.48]	0.093	0.71
	G5 − G6	12	2.37	3.10	[−0.15, 4.90]	0.068	0.76
D	G7 − G8	12	0.63	4.07	[−2.69, 3.94]	1.000	0.15
	G7 − G9	12	0.81	3.72	[−2.22, 3.84]	1.000	0.22
	G8 − G9	12	0.18	1.36	[−0.93, 1.29]	1.000	0.13
E	G10 − G11	12	0.02	2.20	[−1.77, 1.80]	1.000	0.01
	G10 − G12	12	0.92	1.87	[−0.61, 2.44]	0.352	0.49
	G11 − G12	12	0.90	1.73	[−0.51, 2.31]	0.293	0.52
F	G13 − G14	12	0.25	2.46	[−1.75, 2.25]	1.000	0.10
	G13 − G15	12	0.03	0.99	[−0.78, 0.84]	1.000	0.03
	G14 − G15	12	−0.22	2.20	[−2.01, 1.57]	1.000	−0.10

Each observation represents one athlete’s Overall MAPE obtained from the corresponding held-out-athlete fold. Mean differences are expressed in percentage points and were calculated as the first model minus the second model. SD represents the standard deviation of the athlete-level paired differences. Cohen’s *d_z_* was calculated as the mean paired difference divided by the *SD* of the paired differences. Confidence intervals and p values were adjusted using the Bonferroni method within each device group. Group B contained only G2 and therefore had no within-group comparison.

**Table 4:** Bonferroni-adjusted pairwise comparisons of athlete-level Overall MAPE among device groups.

Comparison	n	Mean difference	*SD* of paired differences	Bonferroni-adjusted 95% CI	Adjusted *p*	Cohen’s *d_z_*
A − B	12	4.18	2.70	[1.28, 7.09]	0.003	1.55
A − C	12	−4.92	4.68	[−9.95, 0.12]	0.058	−1.05
A − D	12	1.79	1.92	[−0.28, 3.85]	0.121	0.93
A − E	12	1.65	2.64	[−1.19, 4.50]	0.789	0.63
A − F	12	5.13	2.16	[2.80, 7.45]	<0.001	2.37
B − C	12	−9.10	4.81	[−14.27, −3.93]	<0.001	−1.89
B − D	12	−2.39	2.93	[−5.55, 0.76]	0.246	−0.82
B − E	12	−2.53	3.01	[−5.76, 0.71]	0.212	−0.84
B − F	12	0.94	2.19	[−1.41, 3.30]	1.000	0.43
C − D	12	6.71	3.78	[2.63, 10.78]	0.001	1.77
C − E	12	6.57	3.56	[2.74, 10.41]	<0.001	1.84
C − F	12	10.04	3.46	[6.32, 13.77]	<0.001	2.90
D − E	12	−0.13	1.91	[−2.19, 1.93]	1.000	−0.07
D − F	12	3.34	1.75	[1.45, 5.22]	<0.001	1.91
E − F	12	3.47	2.13	[1.18, 5.76]	0.002	1.63

Each observation represents one athlete-level device-group Overall MAPE value. Mean differences are expressed in percentage points and were calculated as the first group minus the second group. SD represents the standard deviation of the athlete-level paired differences. Confidence intervals and p values were adjusted using the Bonferroni method.

**Table 5:** Bonferroni-adjusted pairwise comparisons of athlete-level MAPE among exercise tasks.

Comparison	n	Mean difference	*SD* of paired differences	Bonferroni-adjusted 95% CI	Adjusted *p*	Cohen’s *d*_z_
T1 − T2	11	0.20	8.52	[−9.63, 10.03]	1.000	0.02
T1 − T3	11	4.22	8.18	[−5.22, 13.66]	1.000	0.52
T1 − T4	11	3.46	6.54	[−4.09, 11.00]	1.000	0.53
T1 − T5	11	2.33	6.98	[−5.73, 10.38]	1.000	0.33
T1 − T6	11	1.42	7.54	[−7.28, 10.12]	1.000	0.19
T2 − T3	11	4.02	4.01	[−0.61, 8.65]	0.116	1.00
T2 − T4	11	3.25	5.00	[−2.52, 9.03]	0.846	0.65
T2 − T5	11	2.12	4.95	[−3.58, 7.83]	1.000	0.43
T2 − T6	11	1.22	4.66	[−4.16, 6.60]	1.000	0.26
T3 − T4	11	−0.76	3.13	[−4.38, 2.85]	1.000	−0.24
T3 − T5	11	−1.89	3.32	[−5.73, 1.94]	1.000	−0.57
T3 − T6	11	−2.80	3.65	[−7.01, 1.42]	0.440	−0.77
T4 − T5	11	−1.13	3.17	[−4.79, 2.53]	1.000	−0.36
T4 − T6	11	−2.04	3.56	[−6.14, 2.07]	1.000	−0.57
T5 − T6	11	−0.91	3.05	[−4.42, 2.61]	1.000	−0.30

Each observation represents one athlete-level task MAPE value. Mean differences are expressed in percentage points and were calculated as the first task minus the second task. SD represents the standard deviation of the athlete-level paired differences. Confidence intervals and p values were adjusted using the Bonferroni method.

For the within-device-group comparisons in **Table 3**, each athlete contributed one Overall MAPE value for each relevant signal combination. Group B contained only G2 and was therefore excluded from the within-device-group inferential analysis. For the device-group comparisons in **Table 4**, each athlete’s group-level value was calculated as the mean Overall MAPE across the models included in that group: Group A, G1 and G3; Group B, G2; Group C, G4–G6; Group D, G7–G9; Group E, G10–G12; and Group F, G13–G15. For the task comparisons in **Table 5**, each athlete’s task-level value was calculated by averaging MAPE across G1–G15 for the corresponding task. Only athletes with complete data across T1–T6 were included in the task analysis (*n* = 11). To maintain a consistent descriptive sample across conditions, Overall MAPE was also summarized using the same 11 athletes; however, the Overall condition was not included in the repeated-measures analysis. For the descriptive agreement assessment of G15 during T3, the mean signed prediction error (measured minus predicted VO_2_) and 95% limits of agreement (mean error ± 1.96 SD) were calculated from the pooled 1-H_Z_ held-out predictions.

Normality was assessed using the Shapiro-Wilk test. Repeated-measures analysis of variance was used to compare prediction performance, with the Greenhouse-Geisser correction applied when the assumption of sphericity was violated. *Post hoc* paired comparisons were adjusted using the Bonferroni method. Mean differences were calculated as the first condition minus the second condition. The SD reported in **Tables 3**-**5** represents the standard deviation of the athlete-level paired differences. Cohen’s *d_z_* was calculated as the mean paired difference divided by the SD of the paired differences. Bonferroni-adjusted 95% confidence intervals and adjusted p values were reported for the pairwise comparisons. Effect sizes for omnibus tests were reported as partial eta squared (η*_p_*^2^). Statistical significance was set at *p* < 0.05.

## 3 Results

### 3.1 Predictive performance across signal combinations and exercise tasks

**Figure 1:**
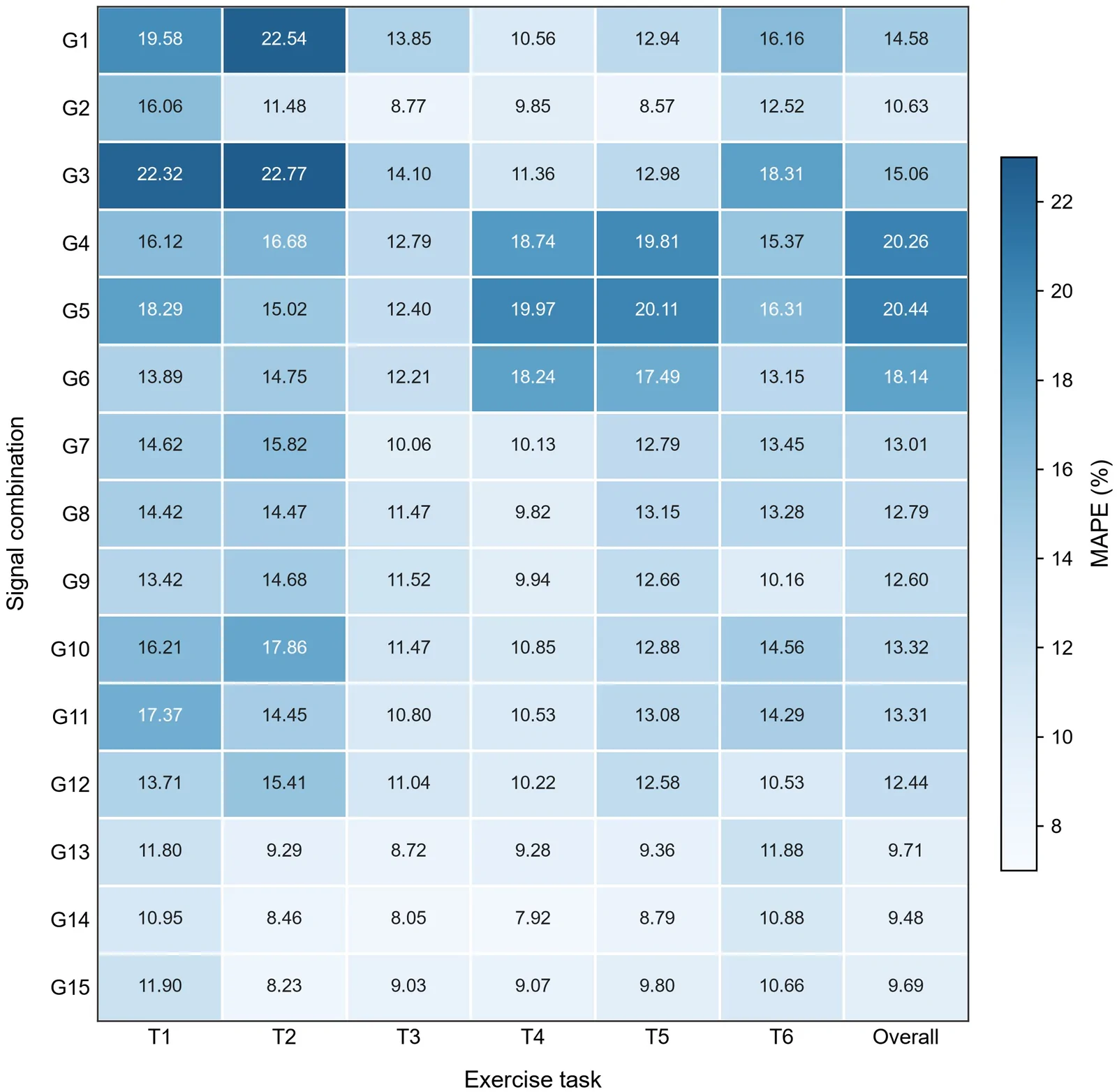
Descriptive MAPE values for the 15 signal combinations across T1–T6 and the overall condition. The overall condition represents descriptive performance from models trained using pooled observations from T1–T6 and is not an additional exercise task. Each cell shows the pooled MAPE (%) for the corresponding model-task condition using the available prediction data.

The predictive performance of the 15 physiological signal combinations across six exercise tasks and the Overall condition is presented in **Table 6** and **Figure 1**. The Overall condition represents descriptive performance derived from models trained using pooled observations from T1–T6 and does not constitute an additional exercise task. Minor variations in sample size (*n* = 11–12) were primarily attributable to occasional signal loss or insufficient signal quality during specific trials. The values presented in **Table 6** and **Figure 1** are descriptive summaries calculated using the available prediction data for each model-task condition.

**Table 6:** Prediction performance of all signal combinations across exercise tasks.

Device group	Signal combination	Task	*n*	Measured VO_2_	Predicted VO_2_	MAE	RMSE	MAPE (%)	*r*
A	G1	T1	12	21.85 ± 6.42	21.43 ± 3.13	4.10	5.55	19.58	0.51
		T2	12	30.36 ± 8.03	29.60 ± 3.40	5.35	6.80	22.54	0.56
		T3	12	34.04 ± 7.03	33.65 ± 4.31	3.45	4.73	13.85	0.76
		T4	12	26.19 ± 7.14	25.82 ± 5.67	2.49	3.09	10.56	0.91
		T5	12	23.71 ± 6.30	23.50 ± 5.20	2.68	3.52	12.94	0.83
		T6	12	29.37 ± 7.93	29.02 ± 5.74	4.05	5.31	16.16	0.74
		Overall	12	25.88 ± 7.55	25.98 ± 6.23	3.23	4.32	14.58	0.82
B	G2	T1	12	21.85 ± 6.42	21.97 ± 4.29	3.29	4.54	16.06	0.71
		T2	12	30.36 ± 8.03	29.70 ± 6.14	2.84	3.76	11.49	0.90
		T3	12	34.04 ± 7.03	34.38 ± 5.64	2.20	3.22	8.77	0.90
		T4	12	26.19 ± 7.14	26.16 ± 6.54	2.27	2.97	9.85	0.91
		T5	12	23.71 ± 6.30	23.69 ± 5.88	1.81	2.51	8.57	0.92
		T6	12	29.37 ± 7.93	29.08 ± 6.32	3.20	4.24	12.52	0.85
		Overall	12	25.88 ± 7.55	25.66 ± 6.77	2.42	3.50	10.63	0.89
A	G3	T1	12	21.85 ± 6.42	21.56 ± 2.34	4.54	5.79	22.32	0.44
		T2	12	30.36 ± 8.03	29.97 ± 3.42	5.39	6.94	22.77	0.51
		T3	12	34.04 ± 7.03	33.37 ± 3.88	3.63	5.26	14.10	0.68
		T4	12	26.19 ± 7.14	25.89 ± 5.61	2.60	3.24	11.36	0.90
		T5	12	23.71 ± 6.30	23.70 ± 5.17	2.68	3.48	12.98	0.83
		T6	12	29.37 ± 7.93	29.11 ± 5.10	4.53	5.83	18.31	0.68
		Overall	12	25.88 ± 7.55	25.81 ± 5.96	3.43	4.65	15.06	0.79
C	G4	T1	11	21.21 ± 6.02	20.80 ± 4.14	3.34	4.54	16.12	0.66
		T2	12	30.36 ± 8.03	30.54 ± 5.06	4.08	5.53	16.68	0.73
		T3	12	34.04 ± 7.03	33.80 ± 4.56	3.39	4.83	12.79	0.73
		T4	11	26.10 ± 7.17	25.26 ± 4.80	4.30	5.39	18.74	0.67
		T5	12	23.68 ± 6.34	23.33 ± 3.98	4.27	5.56	19.81	0.50
		T6	12	29.37 ± 7.93	29.11 ± 5.82	4.03	5.21	15.37	0.76
		Overall	12	25.86 ± 7.59	25.61 ± 5.13	4.65	5.95	20.26	0.63
	G5	T1	11	21.21 ± 6.02	21.07 ± 3.65	3.65	4.84	18.29	0.60
		T2	12	30.36 ± 8.03	30.19 ± 5.71	3.67	4.82	15.02	0.81
		T3	12	34.04 ± 7.03	33.59 ± 4.28	3.35	4.90	12.40	0.73
		T4	11	26.10 ± 7.17	25.72 ± 4.62	4.52	5.51	19.97	0.64
		T5	12	23.68 ± 6.34	23.24 ± 3.38	4.25	5.48	20.11	0.51
		T6	12	29.37 ± 7.93	28.99 ± 5.58	4.17	5.45	16.31	0.73
		Overall	12	25.86 ± 7.59	25.72 ± 4.97	4.60	5.87	20.44	0.64
	G6	T1	11	21.21 ± 6.02	20.82 ± 4.83	2.87	4.16	13.89	0.73
		T2	12	30.36 ± 8.03	30.55 ± 5.94	3.65	4.93	14.75	0.79
		T3	12	34.04 ± 7.03	33.14 ± 5.55	3.48	4.52	12.21	0.78
		T4	11	26.10 ± 7.17	24.34 ± 6.63	4.31	5.54	18.24	0.71
		T5	12	23.68 ± 6.34	23.40 ± 4.24	3.78	4.93	17.49	0.63
		T6	12	29.37 ± 7.93	28.86 ± 6.67	3.57	4.71	13.15	0.81
		Overall	12	25.86 ± 7.59	25.36 ± 6.03	4.23	5.48	18.14	0.70
D	G7	T1	11	21.21 ± 6.02	20.71 ± 4.16	3.04	4.12	14.62	0.73
		T2	12	30.36 ± 8.03	29.54 ± 5.82	4.04	5.34	15.82	0.75
		T3	12	34.04 ± 7.03	33.81 ± 5.80	2.72	3.71	10.06	0.85
		T4	11	26.10 ± 7.17	25.64 ± 6.25	2.35	2.91	10.13	0.92
		T5	12	23.68 ± 6.34	23.39 ± 5.26	2.75	3.45	12.79	0.84
		T6	12	29.37 ± 7.93	29.14 ± 6.53	3.62	4.58	13.45	0.82
		Overall	12	25.86 ± 7.59	25.59 ± 6.66	3.01	3.99	13.01	0.85
	G8	T1	11	21.21 ± 6.02	21.08 ± 4.27	2.96	4.23	14.42	0.71
		T2	12	30.36 ± 8.03	30.03 ± 5.86	3.57	4.71	14.47	0.82
		T3	12	34.04 ± 7.03	33.72 ± 5.17	3.07	4.33	11.47	0.79
		T4	11	26.10 ± 7.17	25.86 ± 6.53	2.27	2.85	9.82	0.92
		T5	12	23.68 ± 6.34	23.50 ± 5.36	2.80	3.66	13.15	0.82
		T6	12	29.37 ± 7.93	29.06 ± 6.08	3.46	4.52	13.28	0.83
		Overall	12	25.86 ± 7.59	25.71 ± 6.61	2.93	3.97	12.80	0.85
	G9	T1	11	21.21 ± 6.02	20.85 ± 4.43	2.81	3.91	13.42	0.76
		T2	12	30.36 ± 8.03	29.66 ± 5.92	3.73	4.80	14.68	0.81
		T3	12	34.04 ± 7.03	32.74 ± 5.72	3.26	4.20	11.52	0.82
		T4	11	26.10 ± 7.17	25.74 ± 6.10	2.32	2.95	9.94	0.92
		T5	12	23.68 ± 6.34	23.50 ± 5.57	2.74	3.48	12.66	0.84
		T6	12	29.37 ± 7.93	29.05 ± 6.99	2.78	3.66	10.16	0.89
		Overall	12	25.86 ± 7.59	25.43 ± 6.71	2.95	3.91	12.60	0.86
E	G10	T1	11	21.21 ± 6.02	20.71 ± 4.09	3.42	4.82	16.22	0.61
		T2	12	30.36 ± 8.03	30.28 ± 4.69	4.33	5.84	17.86	0.69
		T3	12	34.04 ± 7.03	33.47 ± 4.94	3.19	4.45	11.47	0.78
		T4	11	26.10 ± 7.17	25.42 ± 6.06	2.49	3.15	10.85	0.91
		T5	12	23.68 ± 6.34	23.45 ± 5.44	2.77	3.50	12.88	0.84
		T6	12	29.37 ± 7.93	29.16 ± 6.14	3.94	4.94	14.56	0.78
		Overall	12	25.86 ± 7.59	25.59 ± 6.46	3.12	4.12	13.32	0.84
	G11	T1	11	21.21 ± 6.02	21.33 ± 4.10	3.48	4.85	17.37	0.60
		T2	12	30.36 ± 8.03	30.09 ± 5.67	3.63	5.20	14.45	0.77
		T3	12	34.04 ± 7.03	33.82 ± 5.29	2.98	4.27	10.80	0.80
		T4	11	26.10 ± 7.17	25.95 ± 6.23	2.39	3.06	10.53	0.91
		T5	12	23.68 ± 6.34	23.48 ± 5.45	2.78	3.75	13.08	0.81
		T6	12	29.37 ± 7.93	28.94 ± 5.79	3.71	4.85	14.29	0.80
		Overall	12	25.86 ± 7.59	25.72 ± 6.58	3.06	4.16	13.31	0.84
	G12	T1	11	21.21 ± 6.02	20.78 ± 4.64	2.87	3.90	13.71	0.76
		T2	12	30.36 ± 8.03	30.34 ± 6.25	3.80	5.15	15.41	0.77
		T3	12	34.04 ± 7.03	32.84 ± 6.14	3.15	4.11	11.04	0.83
		T4	11	26.10 ± 7.17	25.72 ± 5.85	2.37	2.97	10.22	0.92
		T5	12	23.68 ± 6.34	23.26 ± 5.29	2.72	3.50	12.58	0.84
		T6	12	29.37 ± 7.93	28.80 ± 7.01	2.89	3.75	10.53	0.88
		Overall	12	25.86 ± 7.59	25.51 ± 6.82	2.90	3.90	12.44	0.86
F	G13	T1	11	21.21 ± 6.02	21.74 ± 5.13	2.27	2.98	11.80	0.87
		T2	12	30.36 ± 8.03	30.17 ± 6.93	2.29	3.16	9.29	0.92
		T3	12	34.04 ± 7.03	34.44 ± 5.96	2.33	3.21	8.72	0.89
		T4	11	26.10 ± 7.17	25.60 ± 6.39	2.24	2.93	9.28	0.92
		T5	12	23.68 ± 6.34	23.24 ± 5.89	2.04	2.67	9.36	0.91
		T6	12	29.37 ± 7.93	29.01 ± 7.03	3.25	4.20	11.88	0.85
		Overall	12	25.86 ± 7.59	25.55 ± 6.98	2.28	3.19	9.71	0.91
	G14	T1	11	21.21 ± 6.02	21.52 ± 5.04	2.05	3.01	10.95	0.87
		T2	12	30.36 ± 8.03	30.51 ± 6.79	2.06	2.74	8.46	0.95
		T3	12	34.04 ± 7.03	33.82 ± 6.47	2.15	3.11	8.05	0.90
		T4	11	26.10 ± 7.17	26.07 ± 6.45	1.87	2.45	7.92	0.94
		T5	12	23.68 ± 6.34	23.53 ± 5.87	1.89	2.75	8.79	0.90
		T6	12	29.37 ± 7.93	28.99 ± 6.84	2.92	3.81	10.89	0.88
		Overall	12	25.86 ± 7.59	25.73 ± 7.00	2.21	3.32	9.48	0.90
	G15	T1	11	21.21 ± 6.02	21.88 ± 5.14	2.26	2.97	11.90	0.88
		T2	12	30.36 ± 8.03	30.36 ± 7.27	2.03	2.87	8.23	0.94
		T3	12	34.04 ± 7.03	33.60 ± 6.58	2.45	3.45	9.03	0.88
		T4	11	26.10 ± 7.17	25.65 ± 6.81	2.25	2.92	9.07	0.92
		T5	12	23.68 ± 6.34	23.27 ± 6.01	2.17	2.94	9.80	0.89
		T6	12	29.37 ± 7.93	28.76 ± 6.76	2.91	3.68	10.66	0.89
		Overall	12	25.86 ± 7.59	25.38 ± 7.13	2.33	3.36	9.69	0.90

Minor variations in sample size (*n* = 11–12) were primarily due to occasional signal loss or insufficient signal quality from the muscle-oxygenation sensors during specific trials. The Overall condition represents descriptive performance derived from models trained using pooled observations from T1–T6 and does not constitute an additional exercise task. Measured VO_2_, predicted VO_2_, MAE, and RMSE are expressed in mL·kg^-1^·min^-1^; MAPE is expressed as a percentage.

In the Overall analysis, G13–G15 showed relatively low prediction errors. G14 yielded an MAE of 2.21 mL·kg^-1^·min^-1^, an RMSE of 3.32 mL·kg^-1^·min^-1^, and a MAPE of 9.48%. G13 yielded an MAE of 2.28 mL·kg^-1^·min^-1^, an RMSE of 3.19 mL·kg^-1^·min^-1^, a MAPE of 9.71%, and the highest correlation coefficient among the three combinations (*r* = 0.91). G15 yielded an MAE of 2.33 mL·kg^-1^·min^-1^, an RMSE of 3.36 mL·kg^-1^·min^-1^, and a MAPE of 9.69%.

Across the individual exercise tasks, the lowest task-specific MAPE was observed for G14 during T4 (7.92%), whereas the highest task-specific MAPE was observed for G3 during T2 (22.77%). The complete MAE, RMSE, MAPE, and correlation results for all model-task conditions are provided in **Table 6**.

### 3.2 Performance differences within device groups

**Figure 2:**
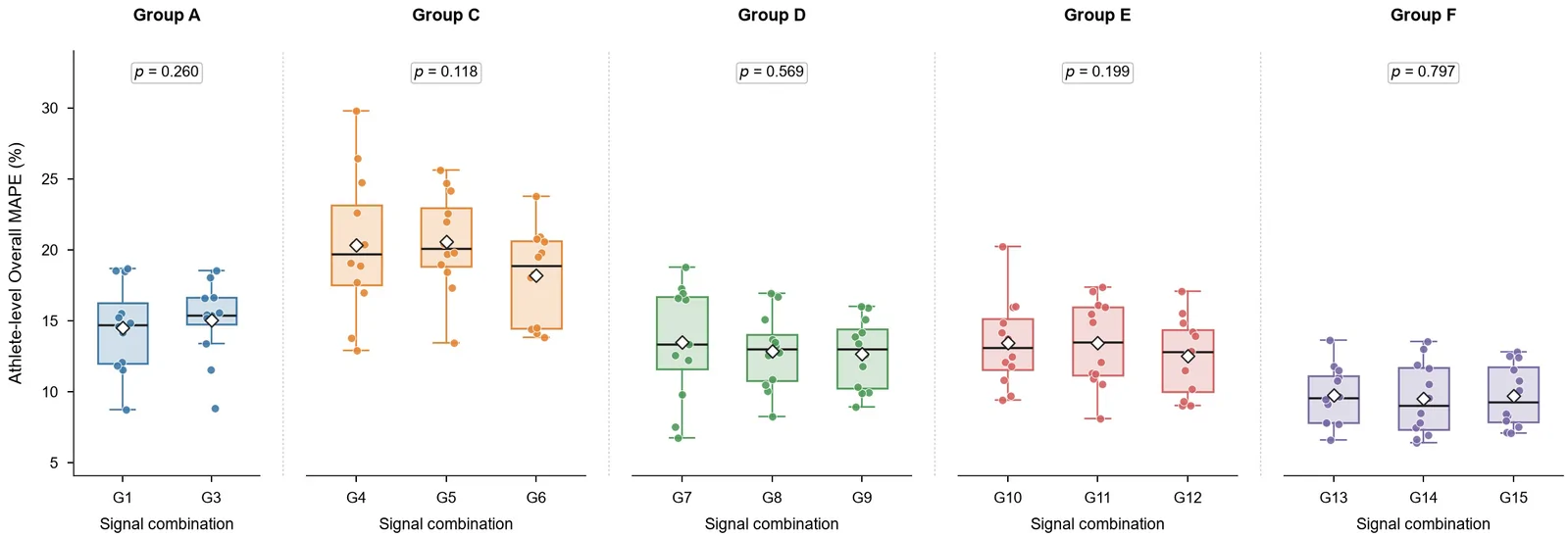
Athlete-level overall MAPE across signal combinations within device groups. Boxplots show the distribution of athlete-level overall MAPE values for each signal combination, with individual points representing held-out-athlete estimates. Boxes indicate the interquartile range, horizontal lines within boxes indicate medians, and diamonds indicate means. The *p* value shown for each device group represents the omnibus repeated-measures test. Group B was not included because it contained only G2.

According to device configuration, the 15 signal combinations were classified into six groups (A–F). **Figure 2** presents the distributions of athlete-level Overall MAPE across signal combinations within each device group. Group B contained only G2 and was therefore excluded from the within-group inferential analysis.

Within-group repeated-measures analyses revealed no significant effect in Group A [*F*(1, 11) = 1.41, *p* = 0.260, partial η*_p_*^2^ = 0.114], Group C [*F*(1.25, 13.70) = 2.70, *p* = 0.118, partial η*_p_*^2^ = 0.197], Group D [*F*(1.17, 12.88) = 0.40, *p* = 0.569, partial η*_p_*^2^ = 0.035], Group E [*F*(1.85, 20.31) = 1.76, *p* = 0.199, partial η*_p_*^2^ = 0.138], or Group F [*F*(1.25, 13.79) = 0.11, *p* = 0.797, partial η*_p_*^2^ = 0.010]. Greenhouse-Geisser-corrected degrees of freedom are reported where applicable.

Consistent with the omnibus tests, none of the Bonferroni-adjusted pairwise comparisons reached statistical significance (**Table 3**). Although G6 showed numerically lower MAPE values than G4 and G5, these differences did not reach statistical significance after adjustment (G4 vs. G6, *p* = 0.093; G5 vs. G6, *p* = 0.068). No significant pairwise differences were observed within Groups A, D, E, or F. Therefore, the numerical differences shown in **Figure 2** should be interpreted descriptively and do not provide sufficient evidence that a specific unilateral, bilateral, or multimodal signal configuration consistently outperformed the other configurations within the same device group.

### 3.3 Performance differences among device groups

**Figure 3:**
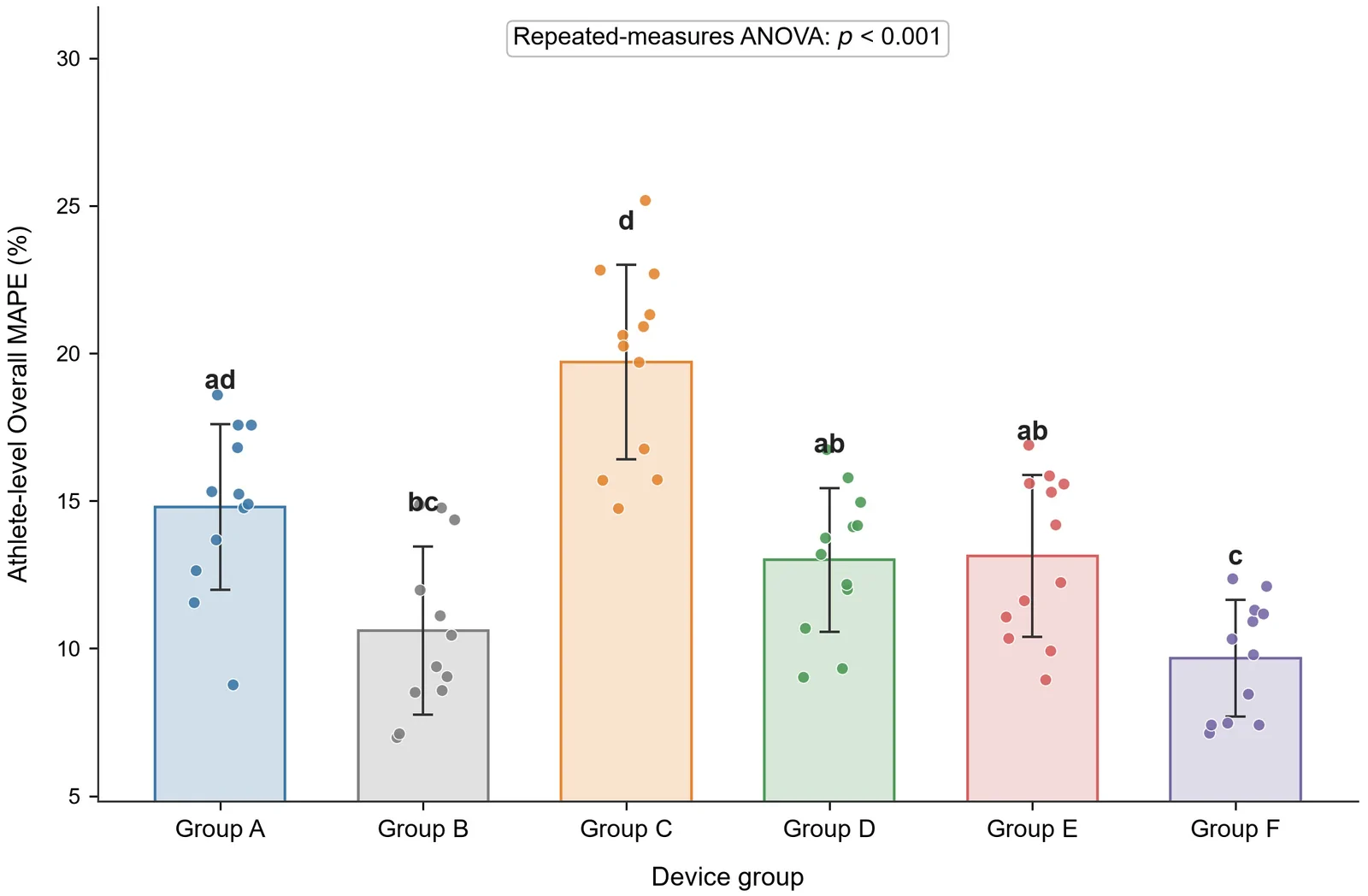
Comparison of athlete-level Overall MAPE among device groups. Bars show means ± SD, and each point represents one athlete’s device-group mean Overall MAPE. Different lowercase letters indicate significant Bonferroni-adjusted pairwise differences; groups sharing a letter did not differ significantly. The omnibus repeated-measures effect was significant (*p* < 0.001).

Significant differences in athlete-level Overall MAPE were observed among the six device groups [*F*(2.42, 26.63) = 32.69, *p* < 0.001, partial η*_p_*^2^ = 0.748], with Greenhouse-Geisser-corrected degrees of freedom reported. Group F showed the lowest mean MAPE (9.66 ± 1.98%), followed by Group B (10.60 ± 2.85%), Group D (13.00 ± 2.44%), Group E (13.13 ± 2.74%), Group A (14.79 ± 2.81%), and Group C (19.70 ± 3.29%) (**Figure 3**).

Bonferroni-adjusted pairwise comparisons showed that Group C had significantly higher MAPE than Groups B, D, E, and F (all adjusted *p* ≤ 0.001), whereas the difference between Groups A and C did not reach statistical significance (*p* = 0.058). Group F showed significantly lower MAPE than Groups A, D, and E (all adjusted *p* ≤ 0.002), but did not differ significantly from Group B (*p* = 1.000). Group B also showed significantly lower MAPE than Group A (*p* = 0.003), but did not differ significantly from Groups D or E. No significant difference was found between Groups D and E (*p* = 1.000). Detailed pairwise results are presented in **Table 4**.

### 3.4 Performance differences across exercise tasks

Athlete-level MAPE was compared across the six exercise tasks using the 11 athletes with complete data for all signal combinations across T1–T6. The Overall condition was presented descriptively using the same 11 complete-case athletes and was not included in the inferential analysis.

**Figure 4:**
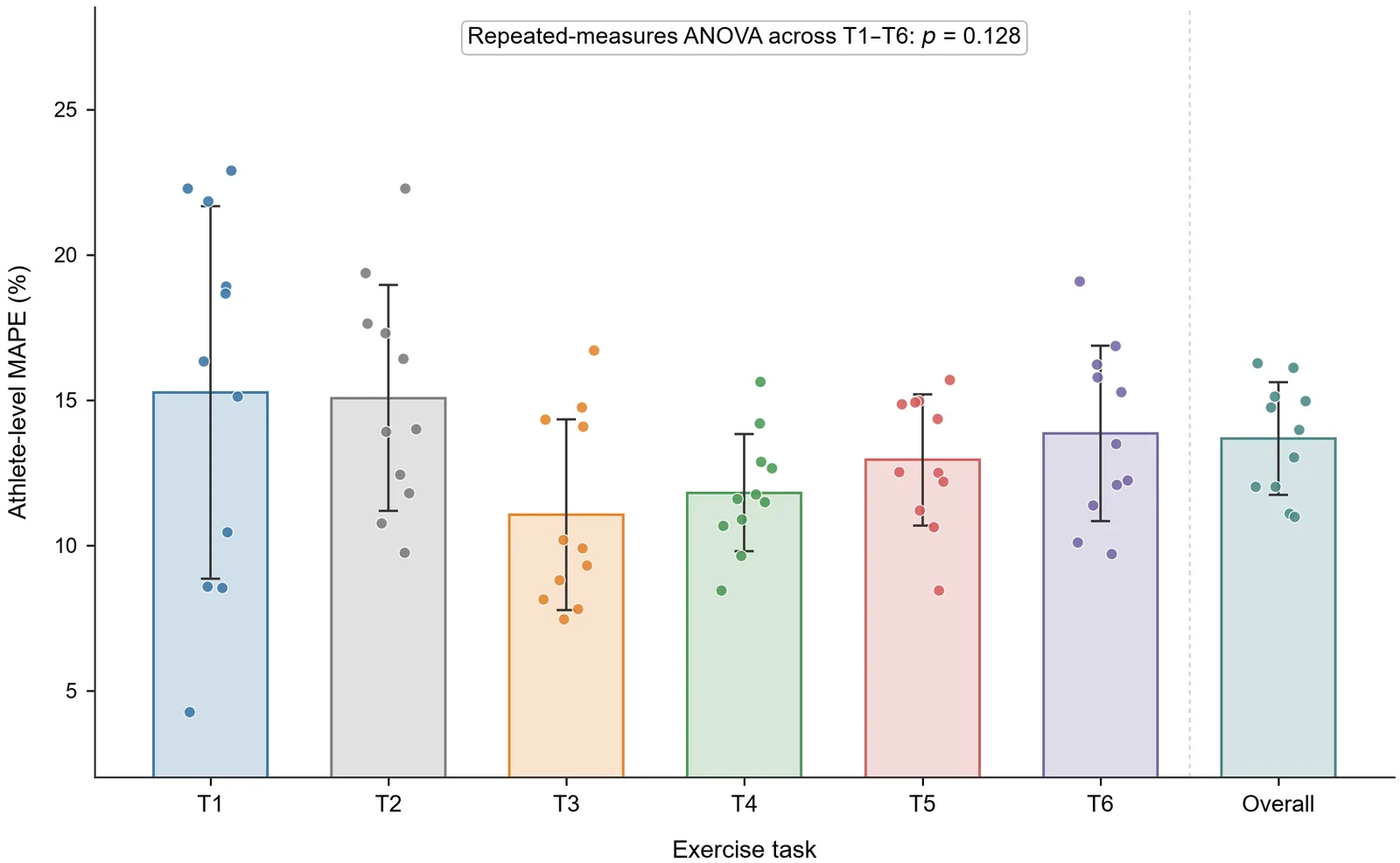
Athlete-level MAPE across exercise tasks. Bars show means ± SD, and each point represents one athlete-level task MAPE value. The omnibus repeated-measures analysis included T1–T6 and was based on 11 complete cases. Overall is shown descriptively using the same 11 athletes and was not included in the inferential analysis.

The repeated-measures analysis revealed no significant effect of exercise task on prediction performance [*F*(2.29, 22.95) = 2.20, *p* = 0.128, partial η*_p_*^2^ = 0.180], with Greenhouse-Geisser-corrected degrees of freedom reported. Numerically, T3 showed the lowest mean MAPE (11.05 ± 3.28%), followed by T4 (11.81 ± 2.01%), T5 (12.94 ± 2.26%), T6 (13.84 ± 3.02%), T2 (15.06 ± 3.89%), and T1 (15.26 ± 6.41%) (**Figure 4**). The descriptive Overall MAPE was 13.67 ± 1.94%.

Consistent with the non-significant omnibus effect, none of the Bonferroni-adjusted pairwise comparisons reached statistical significance (all adjusted *p* > 0.05; **Table 5**). Therefore, the numerical differences across exercise tasks should be interpreted descriptively and do not support a statistically significant ranking of task-specific prediction performance.

### 3.5 Temporal prediction patterns for G15 during T3

**Figure 5:**
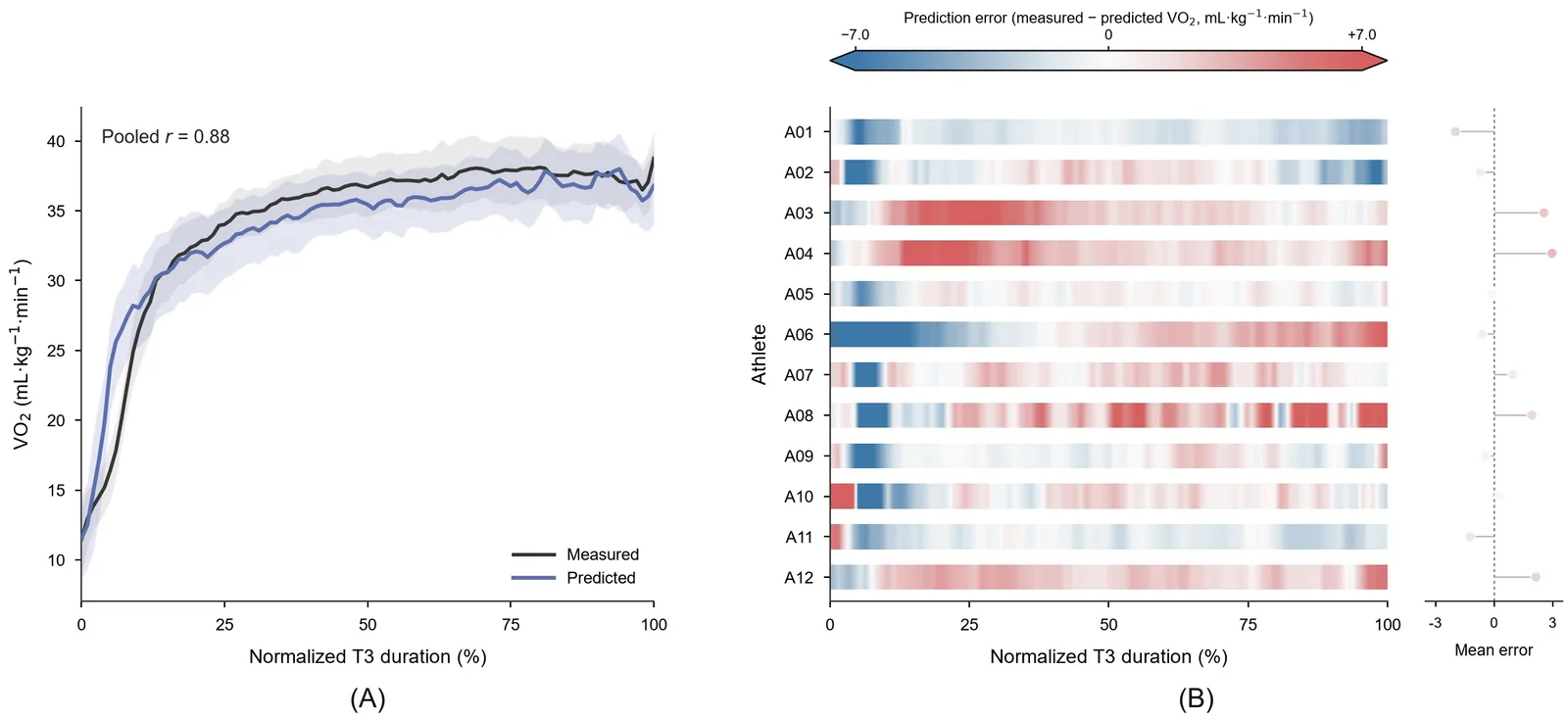
Temporal prediction patterns for G15 during T3. **(A)** Mean measured and predicted VO_2_ trajectories across normalized T3 duration, with shaded bands representing pointwise 95% confidence intervals across athletes. Individual T3 time series were time-normalized to 0–100% of each athlete’s trial duration for visualization. The pooled Pearson correlation coefficient (*r*) was calculated from the original 1-H_Z_ held-out predictions. **(B)** Athlete-specific temporal profiles of prediction error (measured minus predicted VO_2_). Negative values indicate overprediction and positive values indicate underprediction. A01–A12 denote anonymized athletes, and the adjacent dot plot shows the mean signed prediction error for each athlete. Color limits were based on the 95th percentile of absolute prediction errors.

Because G13–G15 showed comparable performance, G15 was selected as a representative fully multimodal configuration because it incorporated smart-shirt-derived cardiorespiratory signals together with bilateral muscle-oxygenation signals. **Figure 5** provides a descriptive illustration of its temporal prediction patterns during T3. At the group level, the predicted VO_2_ trajectory broadly followed the overall temporal pattern of the measured VO_2_ response across the normalized T3 duration (**Figure 5A**). Measured and predicted VO_2_ values from the original pooled 1-H_Z_ observations showed a strong positive correlation (*r* = 0.88). The mean signed prediction error (measured minus predicted VO_2_) was 0.44 mL·kg^-1^·min^-1^, with descriptive 95% limits of agreement ranging from −6.32 to 7.20 mL·kg^-1^·min^-1^.

Athlete-specific prediction-error profiles nevertheless showed variability in both the magnitude and direction of error across athletes and across different phases of T3 (**Figure 5B**). Periods of both overprediction and underprediction were observed, with the magnitude and direction of error varying across the exercise bout. The athlete-level mean signed errors also varied across individuals, indicating that the temporal prediction error was not uniformly distributed among athletes. Overall, G15 captured the general temporal evolution of VO_2_ during T3, while athlete- and phase-specific prediction errors remained.

## 4 Discussion

### 4.1 Multimodal physiological signals for VO_2_ prediction

The present findings indicate that the predictive contribution of multimodal physiological signals depended on the specific input configuration rather than increasing uniformly with the number of signals. In the Overall analysis, G13–G15, which combined smart-shirt-derived cardiorespiratory variables with muscle-oxygenation signals, produced the lowest numerical errors and were statistically comparable, whereas G2, comprising HRt, RR, and device-derived MV without NIRS input, also showed a relatively low prediction error. Previous studies have similarly demonstrated that wearable cardiac and respiratory signals can support dynamic VO_2_ estimation during walking, cycling, and transitions across exercise intensities (Alzamer et al., 2021; Beltrame et al., 2017; Zignoli et al., 2020). Cardiopulmonary variables such as heart rate, respiratory frequency, and ventilation provide information on systemic responses to changing metabolic demand and have been successfully incorporated into nonlinear models for instantaneous VO_2_ estimation (Amelard et al., 2021; Wang et al., 2022). In contrast, NIRS-derived SmO_2_ and HHb primarily characterize local skeletal-muscle oxygen delivery and utilization, providing physiological information that is distinct from whole-body cardiorespiratory responses (Perrey and Ferrari, 2018; Perrey et al., 2024). Taken together, the present results suggest that muscle-oxygenation signals may provide complementary information in some input configurations, but that adding more sensor channels does not necessarily produce a proportional improvement in athlete-level generalization.

### 4.2 Effects of wearable-device configuration on prediction performance

The within-device-group analyses did not identify significant differences among signal combinations in Groups A, C, D, E, or F. Accordingly, the present results do not support a general advantage of the heart-rate armband over the wrist-worn device or a consistent advantage of bilateral over unilateral muscle-oxygenation monitoring. Previous validation studies have shown that the accuracy of optical heart-rate measurements varies according to anatomical placement, exercise mode, and movement intensity, and that heart-rate placement can affect subsequent VO_2_-prediction performance even when respiratory variables are included (Lu et al., 2024; Wang et al., 2017). Therefore, the absence of a significant difference between G1 and G3 should not be interpreted as evidence that the two sensor placements are technically equivalent, but rather that no detectable difference was observed under the present experimental and modeling conditions.

Similarly, bilateral muscle-oxygenation input did not significantly outperform bottom-hand-only or top-hand-only input within any device group. NIRS reflects oxygenation changes within a localized volume of skeletal muscle, and its measurements are influenced by the selected muscle, optode position, adipose-tissue thickness, and spatial heterogeneity in muscle recruitment (Perrey and Ferrari, 2018; Perrey et al., 2024). Bilateral monitoring may therefore capture additional information about inter-arm heterogeneity, but the information from the two arms may also be partly redundant when their metabolic responses are coordinated or when systemic cardiorespiratory signals have already been included (Perrey and Ferrari, 2018; Perrey et al., 2024). The present findings consequently suggest that the contribution of bilateral NIRS was configuration-dependent rather than universally beneficial.

At the device-group level, Groups F and B showed the lowest athlete-level Overall MAPE values and did not differ significantly from one another. Group B contained thoracic heart rate, respiratory rate, and device-derived minute ventilation, whereas Group F combined these smart-shirt-derived cardiorespiratory signals with bottom-hand, top-hand, or bilateral muscle-oxygenation inputs. The strong performance of both groups is consistent with previous evidence that heart rate, breathing frequency, and ventilation provide temporally relevant information for estimating dynamic VO_2_ responses (Amelard et al., 2021; Lu et al., 2024). In contrast, Group C, which relied exclusively on biceps-brachii oxygenation signals, showed the highest group-level error and performed significantly worse than Groups B, D, E, and F. Because pulmonary VO_2_ represents systemic oxygen transport and utilization, whereas NIRS characterizes oxygenation within a limited local tissue region, local muscle oxygenation alone may not adequately represent whole-body metabolic demand (Perrey and Ferrari, 2018; Perrey et al., 2024).

Collectively, these findings indicate that prediction performance depended more on the physiological relevance and coverage of the selected signals than on the number of sensors alone. Cardiorespiratory signals appeared to provide the principal information for VO_2_ prediction, whereas muscle-oxygenation signals may offer complementary value in specific configurations without guaranteeing an additional improvement. This interpretation is consistent with wearable VO_2_ models that have achieved accurate dynamic estimation from cardiorespiratory inputs and with NIRS research emphasizing the site- and context-dependent nature of muscle-oxygenation measurements (Amelard et al., 2021; Perrey and Ferrari, 2018; Perrey et al., 2024).

### 4.3 Influence of exercise task on prediction accuracy

The athlete-level analysis did not identify a significant effect of exercise task on prediction performance across T1–T6, and none of the Bonferroni-adjusted pairwise comparisons reached statistical significance. Although T3 and T4 showed numerically lower mean MAPE values, whereas T1 and T2 showed numerically higher values, these differences should be interpreted as descriptive tendencies rather than evidence of a task-specific performance ranking.

The numerical pattern may partly reflect differences in the temporal characteristics of the exercise protocols. During short-duration maximal exercise, metabolic demand changes rapidly, whereas heart rate and oxygen uptake do not respond synchronously under non-steady-state conditions; this temporal dissociation may weaken the instantaneous relationship between wearable physiological signals and measured VO_2_ (Achten and Jeukendrup, 2003; Bot and Hollander, 2000). Previous wearable-based modeling research has also shown that dynamic VO_2_ estimation across changing exercise intensities benefits from incorporating an extended temporal history, highlighting the importance of accounting for physiological response delays (Amelard et al., 2021).

Because the present multilayer perceptron used contemporaneous physiological inputs without an explicit temporal sequence, it may have had limited capacity to represent delayed cardiorespiratory responses during rapid exercise transitions. However, the non-significant omnibus and pairwise results indicate that this explanation remains tentative and that task type was not a statistically supported determinant of prediction error in the present sample. Future studies incorporating larger samples and sequence-aware models are needed to determine whether exercise duration, transition rate, and intensity systematically influence wearable-based VO_2_ prediction.

### 4.4 Temporal VO_2_ prediction during T3

The temporal analysis of G15 during T3 provides additional insight into model behavior beyond the aggregate prediction-error metrics. At the group level, the predicted VO_2_ trajectory broadly followed the temporal pattern of the measured response throughout the 1000-m test. The strong pooled association between measured and predicted VO_2_ (*r* = 0.88) further indicates that the model captured a substantial proportion of the temporal variation in VO_2_. However, correlation alone does not establish agreement between measured and predicted values. The mean prediction error was 0.44 mL·kg^-1^·min^-1^, with descriptive 95% limits of agreement ranging from −6.32 to 7.20 mL·kg^-1^·min^-1^, indicating that meaningful errors remained at the level of individual observations despite the relatively small average bias (Bland and Altman, 1986).

The athlete-specific temporal error profiles shown in **Figure 5** further demonstrate that prediction errors were not uniformly distributed across individuals or across different phases of T3. Periods of both overprediction and underprediction were evident, suggesting that similar aggregate performance may arise from different temporal error patterns among athletes. These discrepancies may partly reflect the delayed and time-dependent nature of cardiorespiratory responses during changing exercise demands. Because the present multilayer perceptron used contemporaneous physiological inputs without an explicit temporal sequence, it could not directly account for the preceding physiological history contributing to instantaneous VO_2_. Previous wearable-based research has shown that incorporating temporal information can improve dynamic VO_2_ estimation across exercise transitions (Amelard et al., 2021). Sequence-aware modeling and athlete-specific calibration may therefore help reduce the athlete- and phase-specific errors observed in the present study.

### 4.5 Limitations and future research directions

Several limitations should be acknowledged. First, the study included only 12 male, highly trained dragon boat athletes, and the task-level analysis was based on 11 complete cases. The small and relatively homogeneous sample may limit the precision and generalizability of the findings, particularly because cross-validation estimates can be variable in small samples (Varoquaux et al., 2017). Second, occasional signal loss resulted in incomplete data for some athlete–task–model conditions. Third, the held-out athlete also served as the validation dataset during the original modeling procedure; therefore, model tuning and fold-level evaluation were not fully independent, potentially resulting in optimistic performance estimates. In addition, the pooled 1-H_Z_ analyses and temporal patterns shown for G15 during T3 should be interpreted descriptively because repeated observations were nested within athletes.

Future studies should include larger and more diverse samples and use an internal validation set drawn exclusively from the training athletes, nested cross-validation, and independent external validation. Model performance should also be assessed in additional teams and field-based training environments. Further work should compare different NIRS measurement sites because muscle-oxygenation responses are site-dependent (Perrey and Ferrari, 2018). Finally, sequence-aware models incorporating preceding physiological information may better capture the delayed and dynamic characteristics of VO_2_ responses (Amelard et al., 2021).

## 5 Conclusions

This study evaluated machine-learning-based VO_2_ prediction using 15 combinations of wearable cardiorespiratory and muscle-oxygenation signals in dragon boat athletes across six exercise tasks. G13–G15 showed comparably low overall prediction errors and should be regarded as similarly performing multimodal configurations rather than identifying a single combination as uniquely optimal. At the device-group level, Groups F and B showed the lowest athlete-level Overall MAPE values and did not differ significantly from each other. Cardiorespiratory signals appeared to provide useful information for VO_2_ estimation, whereas the contribution of muscle-oxygenation signals depended on the specific input configuration. No significant differences were identified among signal combinations within the same device group, providing no evidence that bilateral muscle-oxygenation monitoring or a greater number of input signals consistently improved prediction performance. Prediction performance also did not differ significantly across exercise tasks. The representative G15 model broadly tracked the temporal evolution of measured VO_2_ during the 1000-m test, although athlete- and phase-specific prediction errors remained. Overall, wearable physiological signals combined with machine-learning methods show potential for non-invasive VO_2_ monitoring in dragon boat athletes, but larger samples, fully independent validation, and more rigorous temporal modeling are required before broader practical application.

## Data Availability

The original contributions presented in the study are included in the article/**Supplementary Material**. Further inquiries can be directed to the corresponding author.
