# Supplementary material for: Prediction of oxygen uptake in dragon boat athletes using machine learning and multimodal physiological signals
Source: Front Physiol. 2026 Sep 7;17:1740055. doi: 10.3389/fphys.2026.1740055 (PMC13595688; doi:10.3389/fphys.2026.1740055)
Supplement: Supplementary file 1 [file Table2.docx]

**The complete prediction result**

|  | **Groups** | **Test** | **N** | **Seconds** | **VO_2_** | **Predicted VO_2_** | **MAE** | **RMSE** | **MAPE（%）** | ***R*^2^** | ***r*** | **Mean Bias** | **LoA Lower** | **LoA Upper** |
| --- | --- | --- | --- | --- | --- | --- | --- | --- | --- | --- | --- | --- | --- | --- |
| A | G1 | T1 | 12 | 574 | 21.85 ± 6.42 | 21.43 ± 3.13 | 4.10 | 5.55 | 19.58 | 0.25 | 0.51 | 0.43 | -10.43 | 11.28 |
|  |  | T2 | 12 | 1638 | 30.36 ± 8.03 | 29.60 ± 3.40 | 5.35 | 6.80 | 22.54 | 0.28 | 0.56 | 0.75 | -12.50 | 14.01 |
|  |  | T3 | 12 | 4006 | 34.04 ± 7.03 | 33.65 ± 4.31 | 3.45 | 4.73 | 13.85 | 0.55 | 0.76 | 0.39 | -8.85 | 9.63 |
|  |  | T4 | 12 | 5802 | 26.19 ± 7.14 | 25.82 ± 5.67 | 2.49 | 3.09 | 10.56 | 0.81 | 0.91 | 0.37 | -5.63 | 6.38 |
|  |  | T5 | 12 | 28860 | 23.71 ± 6.30 | 23.50 ± 5.20 | 2.68 | 3.52 | 12.94 | 0.69 | 0.83 | 0.21 | -6.68 | 7.09 |
|  |  | T6 | 12 | 6575 | 29.37 ± 7.93 | 29.02 ± 5.74 | 4.05 | 5.31 | 16.16 | 0.55 | 0.74 | 0.35 | -10.04 | 10.74 |
|  |  | T7 | 12 | 47455 | 25.88 ± 7.55 | 25.98 ± 6.23 | 3.23 | 4.32 | 14.58 | 0.67 | 0.82 | -0.11 | -8.58 | 8.37 |
|  | G3 | T1 | 12 | 574 | 21.85 ± 6.42 | 21.56 ± 2.34 | 4.54 | 5.79 | 22.32 | 0.19 | 0.44 | 0.29 | -11.05 | 11.62 |
|  |  | T2 | 12 | 1638 | 30.36 ± 8.03 | 29.97 ± 3.42 | 5.39 | 6.94 | 22.77 | 0.25 | 0.51 | 0.39 | -13.19 | 13.97 |
|  |  | T3 | 12 | 4006 | 34.04 ± 7.03 | 33.37 ± 3.88 | 3.63 | 5.26 | 14.10 | 0.44 | 0.68 | 0.67 | -9.56 | 10.90 |
|  |  | T4 | 12 | 5802 | 26.19 ± 7.14 | 25.89 ± 5.61 | 2.60 | 3.24 | 11.36 | 0.79 | 0.90 | 0.30 | -6.02 | 6.63 |
|  |  | T5 | 12 | 28860 | 23.71 ± 6.3 | 23.7 ± 5.17 | 2.68 | 3.48 | 12.98 | 0.69 | 0.83 | 0.01 | -6.82 | 6.84 |
|  |  | T6 | 12 | 6575 | 29.37 ± 7.93 | 29.11 ± 5.1 | 4.53 | 5.83 | 18.31 | 0.46 | 0.68 | 0.25 | -11.16 | 11.66 |
|  |  | T7 | 12 | 47455 | 25.88 ± 7.55 | 25.81 ± 5.96 | 3.43 | 4.65 | 15.06 | 0.62 | 0.79 | 0.06 | -9.05 | 9.18 |
| B | G2 | T1 | 12 | 574 | 21.85 ± 6.42 | 21.97 ± 4.29 | 3.29 | 4.54 | 16.06 | 0.50 | 0.71 | -0.12 | -9.02 | 8.78 |
|  |  | T2 | 12 | 1638 | 30.36 ± 8.03 | 29.7 ± 6.14 | 2.84 | 3.76 | 11.49 | 0.78 | 0.90 | 0.66 | -6.61 | 7.92 |
|  |  | T3 | 12 | 4006 | 34.04 ± 7.03 | 34.38 ± 5.64 | 2.20 | 3.22 | 8.77 | 0.79 | 0.90 | -0.34 | -6.61 | 5.94 |
|  |  | T4 | 12 | 5802 | 26.19 ± 7.14 | 26.16 ± 6.54 | 2.27 | 2.97 | 9.85 | 0.83 | 0.91 | 0.03 | -5.79 | 5.86 |
|  |  | T5 | 12 | 28860 | 23.71 ± 6.3 | 23.69 ± 5.88 | 1.81 | 2.51 | 8.57 | 0.84 | 0.92 | 0.02 | -4.89 | 4.94 |
|  |  | T6 | 12 | 6575 | 29.37 ± 7.93 | 29.08 ± 6.32 | 3.20 | 4.24 | 12.52 | 0.71 | 0.85 | 0.29 | -7.99 | 8.58 |
|  |  | T7 | 12 | 47455 | 25.88 ± 7.55 | 25.66 ± 6.77 | 2.42 | 3.50 | 10.63 | 0.79 | 0.89 | 0.22 | -6.63 | 7.07 |
| C | G4 | T1 | 11 | 531 | 21.21 ± 6.02 | 20.8 ± 4.14 | 3.34 | 4.54 | 16.12 | 0.43 | 0.66 | 0.41 | -8.46 | 9.29 |
|  |  | T2 | 12 | 1638 | 30.36 ± 8.03 | 30.54 ± 5.06 | 4.08 | 5.53 | 16.68 | 0.53 | 0.73 | -0.19 | -11.02 | 10.65 |
|  |  | T3 | 12 | 4006 | 34.04 ± 7.03 | 33.8 ± 4.56 | 3.39 | 4.83 | 12.79 | 0.53 | 0.73 | 0.24 | -9.22 | 9.70 |
|  |  | T4 | 11 | 5321 | 26.1 ± 7.17 | 25.26 ± 4.8 | 4.30 | 5.39 | 18.74 | 0.43 | 0.67 | 0.84 | -9.60 | 11.28 |
|  |  | T5 | 12 | 28379 | 23.68 ± 6.34 | 23.33 ± 3.98 | 4.27 | 5.56 | 19.81 | 0.23 | 0.50 | 0.34 | -10.54 | 11.23 |
|  |  | T6 | 12 | 6575 | 29.37 ± 7.93 | 29.11 ± 5.82 | 4.03 | 5.21 | 15.37 | 0.57 | 0.76 | 0.26 | -9.94 | 10.46 |
|  |  | T7 | 12 | 46450 | 25.86 ± 7.59 | 25.61 ± 5.13 | 4.65 | 5.95 | 20.26 | 0.39 | 0.63 | 0.25 | -11.40 | 11.90 |
|  | G5 | T1 | 11 | 531 | 21.21 ± 6.02 | 21.07 ± 3.65 | 3.65 | 4.84 | 18.29 | 0.35 | 0.60 | 0.14 | -9.34 | 9.62 |
|  |  | T2 | 12 | 1638 | 30.36 ± 8.03 | 30.19 ± 5.71 | 3.67 | 4.82 | 15.02 | 0.64 | 0.81 | 0.17 | -9.28 | 9.62 |
|  |  | T3 | 12 | 4006 | 34.04 ± 7.03 | 33.59 ± 4.28 | 3.35 | 4.90 | 12.40 | 0.52 | 0.73 | 0.46 | -9.10 | 10.01 |
|  |  | T4 | 11 | 5321 | 26.1 ± 7.17 | 25.72 ± 4.62 | 4.52 | 5.51 | 19.97 | 0.41 | 0.64 | 0.38 | -10.40 | 11.15 |
|  |  | T5 | 12 | 28379 | 23.68 ± 6.34 | 23.24 ± 3.38 | 4.25 | 5.48 | 20.11 | 0.25 | 0.51 | 0.44 | -10.26 | 11.14 |
|  |  | T6 | 12 | 6575 | 29.37 ± 7.93 | 28.99 ± 5.58 | 4.17 | 5.45 | 16.31 | 0.53 | 0.73 | 0.38 | -10.28 | 11.04 |
|  |  | T7 | 12 | 46450 | 25.86 ± 7.59 | 25.72 ± 4.97 | 4.60 | 5.87 | 20.44 | 0.40 | 0.64 | 0.14 | -11.36 | 11.64 |
|  | G6 | T1 | 11 | 531 | 21.21 ± 6.02 | 20.82 ± 4.83 | 2.87 | 4.16 | 13.89 | 0.52 | 0.73 | 0.39 | -7.73 | 8.52 |
|  |  | T2 | 12 | 1638 | 30.36 ± 8.03 | 30.55 ± 5.94 | 3.65 | 4.93 | 14.75 | 0.62 | 0.79 | -0.20 | -9.86 | 9.47 |
|  |  | T3 | 12 | 4006 | 34.04 ± 7.03 | 33.14 ± 5.55 | 3.48 | 4.52 | 12.21 | 0.59 | 0.78 | 0.90 | -7.78 | 9.58 |
|  |  | T4 | 11 | 5321 | 26.1 ± 7.17 | 24.34 ± 6.63 | 4.31 | 5.54 | 18.24 | 0.40 | 0.71 | 1.76 | -8.54 | 12.06 |
|  |  | T5 | 12 | 28379 | 23.68 ± 6.34 | 23.4 ± 4.24 | 3.78 | 4.93 | 17.49 | 0.40 | 0.63 | 0.28 | -9.37 | 9.93 |
|  |  | T6 | 12 | 6575 | 29.37 ± 7.93 | 28.86 ± 6.67 | 3.57 | 4.71 | 13.15 | 0.65 | 0.81 | 0.51 | -8.66 | 9.69 |
|  |  | T7 | 12 | 46450 | 25.86 ± 7.59 | 25.36 ± 6.03 | 4.23 | 5.48 | 18.14 | 0.48 | 0.70 | 0.50 | -10.19 | 11.19 |
| D | G7 | T1 | 11 | 531 | 21.21 ± 6.02 | 20.71 ± 4.16 | 3.04 | 4.12 | 14.62 | 0.53 | 0.73 | 0.50 | -7.52 | 8.52 |
|  |  | T2 | 12 | 1638 | 30.36 ± 8.03 | 29.54 ± 5.82 | 4.04 | 5.34 | 15.82 | 0.56 | 0.75 | 0.82 | -9.54 | 11.17 |
|  |  | T3 | 12 | 4006 | 34.04 ± 7.03 | 33.81 ± 5.8 | 2.72 | 3.71 | 10.06 | 0.72 | 0.85 | 0.24 | -7.01 | 7.48 |
|  |  | T4 | 11 | 5321 | 26.1 ± 7.17 | 25.64 ± 6.25 | 2.35 | 2.91 | 10.13 | 0.84 | 0.92 | 0.46 | -5.17 | 6.09 |
|  |  | T5 | 12 | 28379 | 23.68 ± 6.34 | 23.39 ± 5.26 | 2.75 | 3.45 | 12.79 | 0.70 | 0.84 | 0.29 | -6.45 | 7.03 |
|  |  | T6 | 12 | 6575 | 29.37 ± 7.93 | 29.14 ± 6.53 | 3.62 | 4.58 | 13.45 | 0.67 | 0.82 | 0.23 | -8.73 | 9.19 |
|  |  | T7 | 12 | 46450 | 25.86 ± 7.59 | 25.59 ± 6.66 | 3.01 | 3.99 | 13.01 | 0.72 | 0.85 | 0.27 | -7.54 | 8.08 |
|  | G8 | T1 | 11 | 531 | 21.21 ± 6.02 | 21.08 ± 4.27 | 2.96 | 4.23 | 14.42 | 0.51 | 0.71 | 0.13 | -8.15 | 8.42 |
|  |  | T2 | 12 | 1638 | 30.36 ± 8.03 | 30.03 ± 5.86 | 3.57 | 4.71 | 14.47 | 0.66 | 0.82 | 0.32 | -8.90 | 9.55 |
|  |  | T3 | 12 | 4006 | 34.04 ± 7.03 | 33.72 ± 5.17 | 3.07 | 4.33 | 11.47 | 0.62 | 0.79 | 0.32 | -8.14 | 8.78 |
|  |  | T4 | 11 | 5321 | 26.1 ± 7.17 | 25.86 ± 6.53 | 2.27 | 2.85 | 9.82 | 0.84 | 0.92 | 0.24 | -5.32 | 5.80 |
|  |  | T5 | 12 | 28379 | 23.68 ± 6.34 | 23.5 ± 5.36 | 2.80 | 3.66 | 13.15 | 0.67 | 0.82 | 0.18 | -6.99 | 7.35 |
|  |  | T6 | 12 | 6575 | 29.37 ± 7.93 | 29.06 ± 6.08 | 3.46 | 4.52 | 13.28 | 0.68 | 0.83 | 0.31 | -8.53 | 9.14 |
|  |  | T7 | 12 | 46450 | 25.86 ± 7.59 | 25.71 ± 6.61 | 2.93 | 3.97 | 12.80 | 0.73 | 0.85 | 0.15 | -7.63 | 7.93 |
|  | G9 | T1 | 11 | 531 | 21.21 ± 6.02 | 20.85 ± 4.43 | 2.81 | 3.91 | 13.42 | 0.58 | 0.76 | 0.36 | -7.28 | 8.01 |
|  |  | T2 | 12 | 1638 | 30.36 ± 8.03 | 29.66 ± 5.92 | 3.73 | 4.80 | 14.68 | 0.64 | 0.81 | 0.70 | -8.62 | 10.02 |
|  |  | T3 | 12 | 4006 | 34.04 ± 7.03 | 32.74 ± 5.72 | 3.26 | 4.20 | 11.52 | 0.64 | 0.82 | 1.30 | -6.53 | 9.13 |
|  |  | T4 | 11 | 5321 | 26.1 ± 7.17 | 25.74 ± 6.1 | 2.32 | 2.95 | 9.94 | 0.83 | 0.92 | 0.36 | -5.37 | 6.09 |
|  |  | T5 | 12 | 28379 | 23.68 ± 6.34 | 23.5 ± 5.57 | 2.74 | 3.48 | 12.66 | 0.70 | 0.84 | 0.17 | -6.64 | 6.99 |
|  |  | T6 | 12 | 6575 | 29.37 ± 7.93 | 29.05 ± 6.99 | 2.78 | 3.66 | 10.16 | 0.79 | 0.89 | 0.31 | -6.83 | 7.46 |
|  |  | T7 | 12 | 46450 | 25.86 ± 7.59 | 25.43 ± 6.71 | 2.95 | 3.91 | 12.60 | 0.74 | 0.86 | 0.43 | -7.19 | 8.04 |
| E | G10 | T1 | 11 | 531 | 21.21 ± 6.02 | 20.71 ± 4.09 | 3.42 | 4.82 | 16.22 | 0.36 | 0.61 | 0.50 | -8.91 | 9.91 |
|  |  | T2 | 12 | 1638 | 30.36 ± 8.03 | 30.28 ± 4.69 | 4.33 | 5.84 | 17.86 | 0.47 | 0.69 | 0.08 | -11.38 | 11.53 |
|  |  | T3 | 12 | 4006 | 34.04 ± 7.03 | 33.47 ± 4.94 | 3.19 | 4.45 | 11.47 | 0.60 | 0.78 | 0.57 | -8.08 | 9.23 |
|  |  | T4 | 11 | 5321 | 26.1 ± 7.17 | 25.42 ± 6.06 | 2.49 | 3.15 | 10.85 | 0.81 | 0.91 | 0.68 | -5.34 | 6.70 |
|  |  | T5 | 12 | 28379 | 23.68 ± 6.34 | 23.45 ± 5.44 | 2.77 | 3.50 | 12.88 | 0.70 | 0.84 | 0.22 | -6.61 | 7.06 |
|  |  | T6 | 12 | 6575 | 29.37 ± 7.93 | 29.16 ± 6.14 | 3.94 | 4.94 | 14.56 | 0.61 | 0.78 | 0.21 | -9.47 | 9.88 |
|  |  | T7 | 12 | 46450 | 25.86 ± 7.59 | 25.59 ± 6.46 | 3.12 | 4.12 | 13.32 | 0.71 | 0.84 | 0.27 | -7.80 | 8.33 |
|  | G11 | T1 | 11 | 531 | 21.21 ± 6.02 | 21.33 ± 4.1 | 3.48 | 4.85 | 17.37 | 0.35 | 0.60 | -0.12 | -9.62 | 9.39 |
|  |  | T2 | 12 | 1638 | 30.36 ± 8.03 | 30.09 ± 5.67 | 3.63 | 5.20 | 14.45 | 0.58 | 0.77 | 0.27 | -9.92 | 10.45 |
|  |  | T3 | 12 | 4006 | 34.04 ± 7.03 | 33.82 ± 5.29 | 2.98 | 4.27 | 10.80 | 0.63 | 0.80 | 0.22 | -8.15 | 8.59 |
|  |  | T4 | 11 | 5321 | 26.1 ± 7.17 | 25.95 ± 6.23 | 2.39 | 3.06 | 10.53 | 0.82 | 0.91 | 0.15 | -5.85 | 6.14 |
|  |  | T5 | 12 | 28379 | 23.68 ± 6.34 | 23.48 ± 5.45 | 2.78 | 3.75 | 13.08 | 0.65 | 0.81 | 0.20 | -7.15 | 7.54 |
|  |  | T6 | 12 | 6575 | 29.37 ± 7.93 | 28.94 ± 5.79 | 3.71 | 4.85 | 14.29 | 0.63 | 0.80 | 0.42 | -9.04 | 9.88 |
|  |  | T7 | 12 | 46450 | 25.86 ± 7.59 | 25.72 ± 6.58 | 3.06 | 4.16 | 13.31 | 0.70 | 0.84 | 0.15 | -8.00 | 8.29 |
|  | G12 | T1 | 11 | 531 | 21.21 ± 6.02 | 20.78 ± 4.64 | 2.87 | 3.90 | 13.71 | 0.58 | 0.76 | 0.43 | -7.18 | 8.04 |
|  |  | T2 | 12 | 1638 | 30.36 ± 8.03 | 30.34 ± 6.25 | 3.80 | 5.15 | 15.41 | 0.59 | 0.77 | 0.02 | -10.08 | 10.12 |
|  |  | T3 | 12 | 4006 | 34.04 ± 7.03 | 32.84 ± 6.14 | 3.15 | 4.11 | 11.04 | 0.66 | 0.83 | 1.20 | -6.50 | 8.90 |
|  |  | T4 | 11 | 5321 | 26.1 ± 7.17 | 25.72 ± 5.85 | 2.37 | 2.97 | 10.22 | 0.83 | 0.92 | 0.37 | -5.41 | 6.16 |
|  |  | T5 | 12 | 28379 | 23.68 ± 6.34 | 23.26 ± 5.29 | 2.72 | 3.50 | 12.58 | 0.70 | 0.84 | 0.42 | -6.39 | 7.23 |
|  |  | T6 | 12 | 6575 | 29.37 ± 7.93 | 28.8 ± 7.01 | 2.89 | 3.75 | 10.53 | 0.78 | 0.88 | 0.56 | -6.70 | 7.83 |
|  |  | T7 | 12 | 46450 | 25.86 ± 7.59 | 25.51 ± 6.82 | 2.90 | 3.90 | 12.44 | 0.74 | 0.86 | 0.35 | -7.26 | 7.96 |
| F | G13 | T1 | 11 | 531 | 21.21 ± 6.02 | 21.74 ± 5.13 | 2.27 | 2.98 | 11.80 | 0.76 | 0.87 | -0.53 | -6.28 | 5.22 |
|  |  | T2 | 12 | 1638 | 30.36 ± 8.03 | 30.17 ± 6.93 | 2.29 | 3.16 | 9.29 | 0.85 | 0.92 | 0.19 | -5.99 | 6.37 |
|  |  | T3 | 12 | 4006 | 34.04 ± 7.03 | 34.44 ± 5.96 | 2.33 | 3.21 | 8.72 | 0.79 | 0.89 | -0.39 | -6.64 | 5.85 |
|  |  | T4 | 11 | 5321 | 26.1 ± 7.17 | 25.6 ± 6.39 | 2.24 | 2.93 | 9.28 | 0.83 | 0.92 | 0.50 | -5.16 | 6.16 |
|  |  | T5 | 12 | 28379 | 23.68 ± 6.34 | 23.24 ± 5.89 | 2.04 | 2.67 | 9.36 | 0.82 | 0.91 | 0.44 | -4.72 | 5.59 |
|  |  | T6 | 12 | 6575 | 29.37 ± 7.93 | 29.01 ± 7.03 | 3.25 | 4.20 | 11.88 | 0.72 | 0.85 | 0.36 | -7.84 | 8.56 |
|  |  | T7 | 12 | 46450 | 25.86 ± 7.59 | 25.55 ± 6.98 | 2.28 | 3.19 | 9.71 | 0.82 | 0.91 | 0.32 | -5.91 | 6.54 |
|  | G14 | T1 | 11 | 531 | 21.21 ± 6.02 | 21.52 ± 5.04 | 2.05 | 3.01 | 10.95 | 0.75 | 0.87 | -0.31 | -6.17 | 5.56 |
|  |  | T2 | 12 | 1638 | 30.36 ± 8.03 | 30.51 ± 6.79 | 2.06 | 2.74 | 8.46 | 0.88 | 0.95 | -0.15 | -5.52 | 5.21 |
|  |  | T3 | 12 | 4006 | 34.04 ± 7.03 | 33.82 ± 6.47 | 2.15 | 3.11 | 8.05 | 0.80 | 0.90 | 0.22 | -5.87 | 6.31 |
|  |  | T4 | 11 | 5321 | 26.1 ± 7.17 | 26.07 ± 6.45 | 1.87 | 2.45 | 7.92 | 0.88 | 0.94 | 0.03 | -4.76 | 4.82 |
|  |  | T5 | 12 | 28379 | 23.68 ± 6.34 | 23.53 ± 5.87 | 1.89 | 2.75 | 8.79 | 0.81 | 0.90 | 0.14 | -5.23 | 5.52 |
|  |  | T6 | 12 | 6575 | 29.37 ± 7.93 | 28.99 ± 6.84 | 2.92 | 3.81 | 10.89 | 0.77 | 0.88 | 0.38 | -7.05 | 7.82 |
|  |  | T7 | 12 | 46450 | 25.86 ± 7.59 | 25.73 ± 7.0 | 2.21 | 3.32 | 9.48 | 0.81 | 0.90 | 0.14 | -6.37 | 6.63 |
|  | G15 | T1 | 11 | 531 | 21.21 ± 6.02 | 21.88 ± 5.14 | 2.26 | 2.97 | 11.90 | 0.76 | 0.88 | -0.67 | -6.35 | 5.01 |
|  |  | T2 | 12 | 1638 | 30.36 ± 8.03 | 30.36 ± 7.27 | 2.03 | 2.87 | 8.23 | 0.87 | 0.94 | 0.00 | -5.62 | 5.62 |
|  |  | T3 | 12 | 4006 | 34.04 ± 7.03 | 33.6 ± 6.58 | 2.45 | 3.45 | 9.03 | 0.76 | 0.88 | 0.44 | -6.26 | 7.15 |
|  |  | T4 | 11 | 5321 | 26.1 ± 7.17 | 25.65 ± 6.81 | 2.25 | 2.92 | 9.07 | 0.83 | 0.92 | 0.45 | -5.21 | 6.11 |
|  |  | T5 | 12 | 28379 | 23.68 ± 6.34 | 23.27 ± 6.01 | 2.17 | 2.94 | 9.80 | 0.79 | 0.89 | 0.41 | -5.31 | 6.12 |
|  |  | T6 | 12 | 6575 | 29.37 ± 7.93 | 28.76 ± 6.76 | 2.91 | 3.68 | 10.66 | 0.79 | 0.89 | 0.61 | -6.50 | 7.71 |
|  |  | T7 | 12 | 46450 | 25.86 ± 7.59 | 25.38 ± 7.13 | 2.33 | 3.36 | 9.69 | 0.81 | 0.90 | 0.48 | -6.03 | 6.99 |
